# Ghostman: Augmented Reality Application for Telerehabilitation and Remote Instruction of a Novel Motor Skill

**DOI:** 10.1155/2014/646347

**Published:** 2014-04-15

**Authors:** Winyu Chinthammit, Troy Merritt, Scott Pedersen, Andrew Williams, Denis Visentin, Robert Rowe, Thomas Furness

**Affiliations:** ^1^Human Interface Technology Laboratory Australia (HIT Lab AU), School of Engineering and ICT, University of Tasmania, Launceston, Tas 7250, Australia; ^2^Active Work Laboratory, Faculty of Education, University of Tasmania, Launceston, Tas 7250, Australia; ^3^School of Health Sciences, University of Tasmania, Launceston, Tas 7250, Australia; ^4^Human Interface Technology Laboratory (HIT Lab), University of Washington, Seattle, WA 98195, USA

## Abstract

This paper describes a pilot study using a prototype telerehabilitation system (Ghostman). Ghostman is a visual augmentation system designed to allow a physical therapist and patient to inhabit each other's viewpoint in an augmented real-world environment. This allows the therapist to deliver instruction remotely and observe performance of a motor skill through the patient's point of view. In a pilot study, we investigated the efficacy of Ghostman by using it to teach participants to use chopsticks. Participants were randomized to a single training session, receiving either Ghostman or face-to-face instructions by the same skilled instructor. Learning was assessed by measuring retention of skills at 24-hour and 7-day post instruction. As hypothesised, there were no differences in reduction of error or time to completion between participants using Ghostman compared to those receiving face-to-face instruction. These initial results in a healthy population are promising and demonstrate the potential application of this technology to patients requiring learning or relearning of motor skills as may be required following a stroke or brain injury.

## 1. Introduction

To minimise ongoing disability and its associated costs, rehabilitation following surgery, stroke, or a musculoskeletal injury typically requires a course of frequent consultations with allied health professionals to determine and direct a treatment during the rehabilitation period [1]. Ageing is associated with increased disability. As the population ages the need for rehabilitation services will increase, placing additional stress on health services staff and budgets [2]. In addition, costs associated with transporting patients long distances and associated decreases in productivity, particularly for patients from rural areas, will add to the community burden of delivering appropriate services. This will place increasing stress on health services and consequently therapeutic solutions need to become more flexible in delivery.

Best practice face-to-face instruction involves the therapist describing the movement with focus on key areas, performing the movement observed by the trainee and then the trainee practising the movement while the trainer provides verbal feedback on performance, and in some cases manually assisting the target movement. In this situation it has been demonstrated that facilitation of the patient's movement or motor performance is a critical part of the prescribed exercise [3]. In contrast, the lower end of the therapeutic scale may involve patients only receiving brief instruction in the therapist's office and then being sent home to practice the new skills by themselves with only a printed sheet of verbal instructions provided by the therapist to consult (sometimes with model drawings). Alarmingly, the latter example is the most common and is usually attributed to high patient caseloads and limited availability of specialists concentrated within geographical locations outside of metropolitan areas.

Telerehabilitation combines telecommunication, sensing and display technologies, and computing technologies to enable rehabilitation to be conducted at a distance [4]. A telerehabilitation system can increase the reach of a therapist, by enabling them to deliver instruction and assess patient performance remotely. To facilitate this increase in reach and reduction in cost, a system must allow the therapist to perform these services remotely. That is, by reducing the need for patient travel, the cost of accessing rehabilitation services is reduced. There is also a lower chance of further injury and less discomfort for the patient, which may also reduce the impact on the patient's caregiver. By using technology to measure and assess the patient's performance, less time is needed for assessment and, consequently, the efficiency of the therapist may also be improved. By improving the intensity of therapy sessions, greater functional gains can occur [5].

Video-based approaches allow for the remote delivery of instruction and the monitoring of patient performance [6, 7]. Another approach is to capture patient performance and display it in a virtual environment. Performance capture can be achieved via sensor-based approaches, such as data gloves [8, 9] and electromagnetic trackers [8, 10–12], or vision-based approaches such as a webcam [13] or marker tracking [14–16]. This performance information can be displayed in a completely virtual environment [10] or augmented into the real world [14].

Virtual reality (VR) and augmented reality (AR) are potential methods of delivering rehabilitative health services remotely. Both have been effective in the delivery of finger and hand rehabilitation after stroke [17, 18] while VR has also been shown to result in significant improvements in motor function and laterality index score in chronic stroke patients [19]. VR systems have been effectively implemented in telerehabilitation [20] and for remote training [21]. AR systems have been shown to be capable of measuring task-completion time, compactness of task, and speed of hand movement by capturing the patients' hand movements whilst moving a tangible object [14] or with marker-based tracking [15]. Khademi et al. [16] used haptic feedback in conjunction with AR to measure stiffness in a user's arm.

There is evidence that training outcomes are positive when users utilised a first-person viewpoint [7, 22]. Yang et al. used a VR approach with “ghost” metaphor and a first-person viewpoint. The motions of trainer/trainee were captured and recreated entirely in the virtual environment in which the trainer operated. However, the use of the VR approach prevents the trainer to view the real environment, which raises concerns in safety issues and a lack of ability to view other subtle visual cues in the environment such as other parts of the limbs not being tracked/targeted. Kumagai et al. [7] used an AR approach. While it is rendered with a first-person viewpoint, the trainer/trainee was viewing the scene via external computer monitors, as a result, causing a viewpoint displacement between the physical limbs and displayed limbs. The displacement requires users to perform an additional cognitive step, a hand-eye coordination operation (similar to using a computer mouse to move a cursor on the display screen). Nevertheless, the benefit of the first-person view is still evident and likely due to the fact that there is a more direct and correct transfer of proprioceptive information [22], which leads to the core of our proposed Ghostman Design.

## 2. Ghostman Design

This paper discusses proof of concept of our proposed telerehabilitation system, called “Ghostman.” Ghostman is a wearable visual augmentation system in egocentric view through which users can observe their own movement being overlaid with a “ghost” image of the instructor's body in real time. Unlike Yang and Kim [22], the Ghostman uses an AR approach, in which the viewing of the real-world environment is preserved. This allows the users to “inhabit” the other's viewpoint, in a technique we call* inhabiting visual augmentation*, illustrated in Figure 2. The use of AR technology enables Ghostman to closely match sensory modalities of the user such as correct and natural visual cues. The Ghostman makes use of a wearable display, a head mounted display (HMD), which helps minimize the viewpoint displacement between the rendered limbs and the actual limbs. By wearing an HMD, trainees can intuitively mimic the movements of the trainer by observing both their own and the trainer's movements simultaneously through the use of colocated overlaying AR images. An HMD with a pair of inbuilt stereoscopic cameras is connected to a desktop computer, which processes and renders the video, as well as providing network communication.

**Figure 1:**
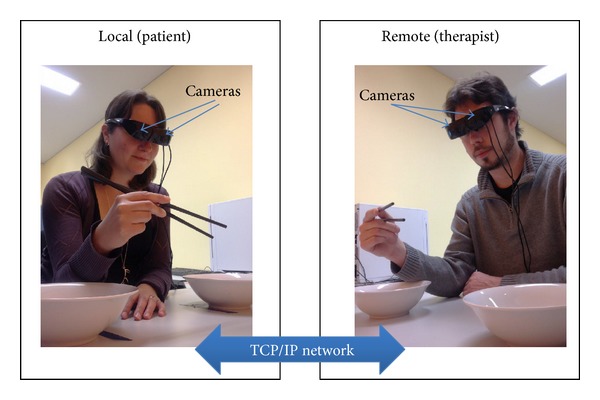
Ghostman setup.

**Figure 2:**
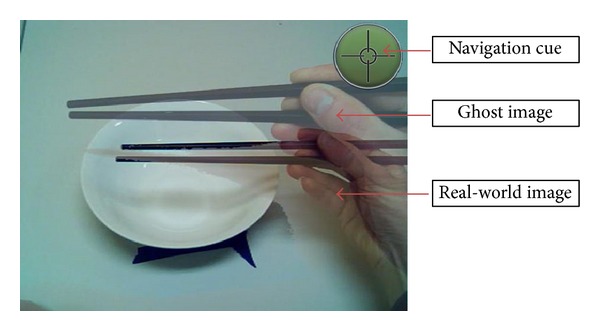
Inhabiting visual augmentation.

Ghostman consists of two subsystems: one is operated by a trainee (patient) and the other by a trainer (therapist), as illustrated in Figure 1. The two subsystems communicate over the internet network, which enables Ghostman to be applied remotely in telepresence applications. Each of the Ghostman subsystems consists of an AR HMD (Vuzix 920AR) that contains a pair of 640 × 480 liquid crystal displays (LCD), a pair of 640 × 480 video cameras, and 3 degrees-of-freedom (DoF) orientation sensors (pitch, yaw, and roll). Each camera is located directly in front of the LCD for each eye minimizing the eye displacement between the display viewpoint and the camera viewpoint, allowing the user to effectively “see through” the HMD with a video see-through AR view. A key design for Ghostman is its ability to visually align the viewpoints of the two HMDs. In order to achieve this posture alignment, one would have to capture the complete movement (6 DoF: 3 orientations and 3 translations) of the heads and hands of both trainer and trainee. However, in this initial study, our aim was to study the performance of a given task with a focus on using the inhabiting visual augmentation technique. Therefore, we decided to limit our task with only orientation head movement to simplify our setup; as a result, our Ghostman proof-of-concept system used only the HMD inbuilt orientation sensor to generate a navigation cue (shown at the top right-handed corner in Figure 2) within the HMD display to allow the trainer to align his head orientation with the trainee's prior to the instructions being given. Furthermore, in order to properly visually align the body parts of the trainer and the trainee, we would have to rescale the overlaid remote limb (depending upon if it is a trainer or trainee) to match the scale of the local limb (undistorted) prior to the overlaying process. The rescaling process is a complicated process, which requires the system to estimate the size of the limbs (e.g., the length of forearm, fingers, and position of elbow) of both trainer/trainee and then rescale the remote limb to match the local limb in real time. However, the focus of this initial study is on the effectiveness of using an inhabiting visual augmentation technique; consequentially, we simplified the setup by assuming the size of the limb (i.e., hand) is the same across all users and therefore there is no rescaling required. It is worth noting that this limb size assumption is not detrimental to this pilot study, as the only visible limb is the hand and lower part of the forearm; thus the effect of rescaling is very small.

Learning by imitation is a key motor learning strategy that has been used previously to evaluate telerehabilitation systems [23]. With Ghostman, the trainee can learn the movements of the trainer by* simultaneously* observing both his/her own and the trainer's movements through the use of real-time overlaying images. Furthermore, Ghostman works in reciprocal fashion allowing the trainer to provide corrective movement feedback in real time.

Ghostman provides a unique environment in delivering movement instructions in egocentric view with a method of integrating description (audio), performance/practice (visual), and assistance/correction (evaluation). With its real-time capability, Ghostman also has the advantage of having the timing of movements as a natural feature of the system, overcoming one of the obstacles when learning a new skill. Therefore, Ghostman might provide an alternative solution in providing therapeutic instructions where more traditional face-to-face methods are difficult to negotiate.

For the cost analysis, each Ghostman system costs approximately $3,000 (AUD) to implement with current hardware. The current cost may not really be suitable for large-scale deployment to patients' home but could be more practical to a remote healthcare community facility where it would only require patients to travel for a short distance.

## 3. Pilot Study

To prove the concept of system a pilot study was conducted to determine the effectiveness of Ghostman in comparison to a best practice method used by physio- and occupational therapists to deliver a complex motor learning sequence to patients. A key component of rehabilitation is the teaching of simple motor skills. The teaching of these skills requires time and expertise of a therapist. The availability and cost of these demands are leading to the use of a telerehabilitation model to reach a wider population of potential clients. The results of this study might provide valuable information regarding the effectiveness of this innovation for motor skill learning, with important implications for the delivery of therapy in an e-health environment.

The aim of this study was to determine the effectiveness of using Ghostman in assisting individuals to learn to perform a novel motor skill using their dominant hand (manipulating chopsticks). The use of chopsticks is a task that can be described as a novel skill that can be learnt within a few minutes and can lead to various levels of expertise. Due to the limitations of the field of view of the Ghostman HMD's cameras, this task was deemed suitable for instructional purposes.

We hypothesise that novice individuals, who use Ghostman to shadow a skilled performer in real time, will be as effective in learning chopstick manipulation technique as individuals who will be similarly trained using a traditional therapeutic method of observing and receiving feedback from a skilled performer in a face-to-face clinical environment. Thus, we tested the null hypothesis with the aim to accept this hypothesis demonstrating that the two types of service delivery are not significantly different in terms of motor learning a novel skill. Due to the limited availability of rehabilitation patients we chose to conduct this pilot study on a healthy population using a convenient sample to provide data for proof of concept of this telerehabilitation.

## 4. Experiment Design

A randomised controlled pilot study was conducted to evaluate the efficacy of the Ghostman prototype as a tool for remote teaching of a novel motor skill using chopsticks. Participants were randomised to receive one teaching session with a skilled instructor delivering the lesson via traditional face-to-face interaction or delivering the lesson via an inhabiting visual augmentation system (Ghostman).

### 4.1. Inclusion Criteria

Adult participants were self-identified as right-handed, as the skilled instructor was right-handed. All participants, who were unfamiliar with using chopsticks (≤ once per year), were recruited through the use of flyers advertising the study.

### 4.2. Exclusion Criteria

Individuals with previously diagnosed dementia or who were unable to comprehend English, individuals with neurological disorders that may affect their ability to learn motor skills, and individuals who had any other conditions preventing use of their right hand were excluded from the study.

### 4.3. Protocol

**Figure 3:**
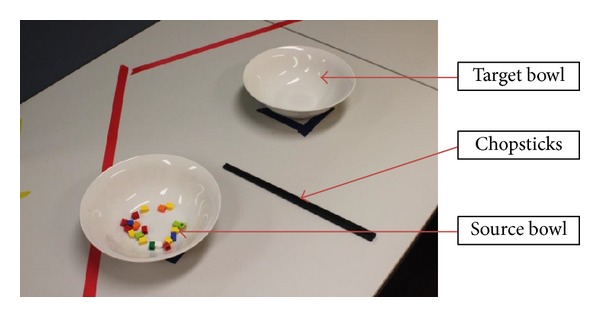
Experiment setup.

Degree of handedness was assessed using a widely used and validated inventory [24]. The testing protocol was designed to follow standard motor learning experiment principles that separate actual skill learning from performance improvements through the use of retention tests [25]. The protocol involved a 7-minute training session and four identical performance tests that were performed at four different sequential times: prior to training (pretest), 5 minutes after training (posttest), 24 hours after training (retention 1), and 7 days after training (retention 2). In each of the tests, participants were seated in front of two identical shallow bowls at a distance of 30 cm from the edge of the table where the participant seated (Figure 3—experiment setup). The source bowl was placed 15 cm to the left side of the participant's midline (xiphoid process) and contained 20 small plastic blocks, all of similar size. The target bowl, which was placed 15 cm to the right side of the participant's midline, was initially empty. Participants were presented with a pair of chopsticks and instructed to transfer all the blocks one at a time to the target bowl. The instructor replaced all dropped pieces back into the source bowl. Total skill errors, the primary dependent variable, were defined as any drops (either within the source bowl or in transit between the two bowls) or gripping errors within the source bowl. The number of skill errors made during each test session was recorded. When blocks were dropped in transit, the instructor collected the errant piece and placed it in the source bowl by hand while the participant continued with the task by attempting to move the next piece. Task completion time, the secondary dependent variable, was measured using a stopwatch to record the time taken to successfully transfer 20 blocks from the source bowl to the target bowl.

Both groups received standardised training from the same expert instructor. The only difference was the method of delivery: Ghostman or face-to-face. The training sessions commenced with instruction on how to hold chopsticks, followed by how to pick up objects with chopsticks. Participants then proceeded to perform a series of practice exercises using blocks of various sizes and received ongoing feedback about their performance from the instructor continuously throughout the seven-minute training session. No feedback was provided during the testing sessions. Ghostman participants were located within the same room as the instructor who was concealed behind a screen, whereas the face-to-face participants had the instructor sitting next to the participant for the duration of the training session. Video recordings of the hand movement and a top view of the test area (i.e., table, bowls) were made of each testing and training session for later analysis.

A user experience questionnaire was provided to assess user perceptions of the instruction methods. Participants were provided a questionnaire to rate their perceptions of the training methods. The five following statements were presented and answered using a 5-point Likert scale, with anchors of 5 corresponding to strong agreement with the statement and 1 indicating strong disagreement with the statement:

1. the instructions I was given were easy to follow,
2. the instructions helped me learn how to use chopsticks,
3. the instructor clearly showed me how to hold the chopsticks,
4. the training programme helped me to learn how to use chopsticks,
5. I feel I am better able to use chopsticks than before the study.

### 4.4. Data Analysis

Demographic data were compared at baseline using independent samples* t*-tests. Questionnaire data were analysed using independent samples* t*-tests to determine any group differences. A 2 (group) X 4 (test) mixed design ANOVA with repeated measures on the last factor was used to test for significant differences for the two dependent variables (total skill error and task completion time) separately, with an alpha level set at 0.05. All statistics were analyzed using IBM SPSS Statistics package version 22. Descriptive statistics were reported as means and standard deviations.

## 5. Results

Preliminary data were collected from 12 participants (6 Ghostman/6 face-to-face), except for the questionnaire data where data were obtained for only five Ghostman participants. There were no differences between the two groups for any variable at baseline (Table 1).

**Figure 4:**
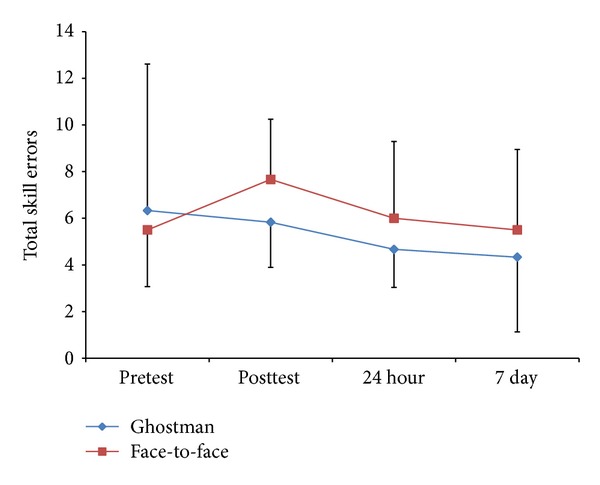
Group by test descriptive statistics (mean ± standard deviation) for total skills errors (frequency count).

Regarding the primary dependent variable, there was no significant interaction (*F*(3,30) = 0.55, *P* = 0.65) or main effects for group (*F*(1,10) = 0.40, *P* = 0.54) or test (*F*(3,30) = 1.00, *P* = 0.40) for total skill errors (Figure 4).

As illustrated in Figure 4, the Ghostman group improved their total skill errors throughout the study as can be interpreted in the mean values from pretest (*M* = 6.33 ± 6.28) to posttest (*M* = 5.83 ± 1.94) to 24-hour retention (*M* = 4.67 ± 1.63) to seven-day retention (*M* = 4.33 ± 3.20), whereas the face group got worse from pretest (*M* = 5.50 ± 2.43) to posttest (M = 7.67 ± 2.58), showed slight improvements after 24 hours (*M* = 6.00 ± 3.29), and finally returned to baseline performance after seven days (*M* = 5.50 ± 3.45) although none of these differences were significant.

**Figure 5:**
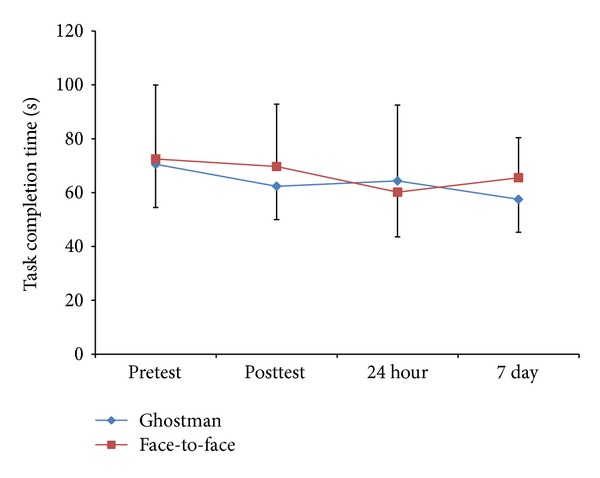
Group by test descriptive statistics (mean ± standard deviation) for task completion time (seconds).

Similarly, for the task completion time dependent variable there was no significant interaction (*F*(3,30) = 0.85, *P* = 0.48) or main effects for group (*F*(1,10) = 0.11, *P* = 0.75) or test (*F*(3,30) = 2.23, *P* = 0.10) (Figure 5). The detail data of Figure 5 are as follows: pretest (Ghostman: *M* = 70.50 ± 16.05, face-to-face: *M* = 72.50 ± 27.42), posttest (Ghostman: *M* = 62.33 ± 12.37, face-to-face: *M* = 69.67 ± 23.19), 24-hour retention (Ghostman: *M* = 64.33 ± 28.12, face-to-face: *M* = 60.17 ± 16.57), and 7-day retention (Ghostman: *M* = 57.50 ± 12.29, face-to-face: *M* = 65.50 ± 14.90)

Finally, there were no differences between groups for any of the Likert scale statements regarding user perceptions of the training methods (Table 2).

## 6. Discussion

The primary outcome of this study (Figures 4 and 5) demonstrated that Ghostman is as effective, in terms of reduction in skill errors and improvements in task completion time, as current best practice face-to-face instruction for learning a novel skill (null hypothesis). Moreover, from the user experience questionnaires (Table 2), participants also felt Ghostman training was as effective as face-to-face training. This provided early evidence that the inhabiting visual augmentation (Ghostman) could be an effective technique for motor learning in a telerehabilitation context. As this is the first study of its kind using telerehabilitation to test the learning of novel skills and there is no previous data for comparison, this pilot study provides promising results for future studies.

Previously, home-based rehabilitation has been demonstrated to be more cost effective than hospital-based rehabilitation [26]. Traditionally, both forms of rehabilitation involve colocation of a therapist and a patient in the same setting, which involves costs associated with transport of the patient/therapist to the setting. The results of the current study indicate that Ghostman is an effective learning tool, which provides further support for the efficacy of a telerehabilitation approach. In addition, telerehabilitation has real potential to reduce cost of rehabilitation delivery by reducing time and travel-related expenses for practitioners and patients alike. However, the cost effectiveness of telerehabilitation delivery has yet to be established [27]. Moreover, the Ghostman telerehabilitation system requires further, larger-scale investigations into the efficacy of this system with clinical populations requiring physical rehabilitation, such as stroke patients or those suffering Parkinson's disease.

### 6.1. Limitations

Caution in interpreting these data is warranted due to the small, convenient sample size used in this study (*n* = 12). As a result, it might be suggested that the lack of statistically significant difference in outcomes between the two training methods might be due to the study being underpowered and thereby making Ghostman appear to be as effective a learning tool as traditional methods. To test the theory that the study was underpowered thereby making Ghostman appear more effective than it is, we conducted post hoc power calculations on the data obtained in this study. These analyses demonstrated that, on the basis of existing data, a total of 508 participants would be required to yield a statistically significant difference in changes in error rate, while 840 participants would be required to produce a statistically significant difference in changes in time to complete the task. In addition, there were greater mean improvements in learning (24-hour and 7-day retention tests) as identified by reductions in skill errors and task completion time when using Ghostman indicating that obtaining a sample size of that projected magnitude would be likely to demonstrate Ghostman to be a more effective learning tool than face-to-face instruction. Another limitation of the study was the training period. Training consisted of a single 7-minute session, regardless of the group. This may not have been a long enough exposure to produce significant improvements in participants. However, this brief amount of training time is consistent with the instruction time typically utilized by therapists when first meeting with new patients. Finally, participants that have been used in this research have been drawn from a healthy population. As such, it is difficult to claim that the technique is valid without examining its efficacy with participants that are currently completing a course of rehabilitation.

## 7. Conclusions

This paper describes our proposed telerehabilitation system (Ghostman) and a pilot study using Ghostman for remotely teaching a novel motor skill. Findings from the pilot study indicated that Ghostman is as effective for motor learning, in terms of reduction in skill errors and improvements in task completion time, as the current best practice face-to-face training. This suggests that Ghostman could be an effective technique for telerehabilitation and for remote instruction of novel motor skill learning applications by physio- and occupational therapists. Given the difficulties that rural and remote communities experience in gaining face-to-face access to health professionals, this outcome holds promise for future development of this technology.

While the early results are encouraging, further development of the Ghostman system and larger-scale studies are required to determine its efficacy in telerehabilitation context. The future development on the current Ghostman system will address the following three main areas: (1) the limited field of view of the camera and of the display (HMD), (2) the rescaling of the remote user's limb (to match with the (undistorted) local limb), and (3) the reduction of the unit cost for large-scale deployment. With these technical improvements, the Ghostman system can then be tested in a large group of participants with more comprehensive case studies that includes expanding ranges of the user movement and working with full-bodied tasks. Ultimately, this would provide valid evidence that the system is ready for real patient trials.

## Figures and Tables

**Table 1:** Comparison of demographic data between the treatment groups (mean ± standard deviation).

Variable	Ghostman	Face-to-face	Significance (*P*)
Gender (M/F)	4/2	4/2	
Age (years)	36.7 ± 11.8	42.2 ± 14.7	0.49
Experience (previous uses)	0.2 ± 0.4	1.0 ± 1.5	0.23
Right handedness (%)	74.1 ± 23.6	93.6 ± 9.9	0.09

**Table 2:** Group by statement descriptive statistics (mean ± standard deviation) for questionnaire data (5-point Likert scale).

	Ghostman (*n* = 5)	Face-to-face (*n* = 6)	Significance (*P*)
Statement 1	4.60 ± 0.55	4.67 ± 0.52	0.84
Statement 2	4.20 ± 0.84	4.50 ± 0.55	0.51
Statement 3	4.80 ± 0.45	4.83 ± 0.41	0.90
Statement 4	4.40 ± 0.55	4.17 ± 0.75	0.57
Statement 5	4.60 ± 0.55	4.17 ± 0.98	0.38
